# Tris(1-ethyl-3-methyl­imidazolium) hexa­bromidoeuropate(III)

**DOI:** 10.1107/S1600536808018382

**Published:** 2008-06-21

**Authors:** Michael Pellens, Ben Thijs, Kristof Van Hecke, Luc Van Meervelt, Koen Binnemans, Peter Nockemann

**Affiliations:** a Laboratory of Coordination Chemistry, Department of Chemistry, Katholieke Universiteit Leuven, Celestijnenlaan 200F bus 2404, B-3001 Leuven, Belgium; b Laboratory of Biomolecular Architecture, Department of Chemistry, Katholieke Universiteit Leuven, Celestijnenlaan 200F bus 2404, B-3001 Leuven, Belgium

## Abstract

The crystal structure of the title compound, (C_6_H_11_N_2_)_3_[EuBr_6_], consists of 1-ethyl-3-methyl­imidazolium cations and centrosymmetric octa­hedral hexa­bromido­europate anions. The [EuBr_6_]^3−^ anions are located at the corners and face-centres of the monoclinic unit cell. Characteristic hydrogen-bonding inter­actions can be observed between the bromide anions and the acidic H atoms of the imidazolium cations.

## Related literature

For related literature, see: Arenz *et al.* (2005 ▶); Binnemans (2007 ▶); Chaumont & Wipff (2003 ▶); Driesen *et al.* (2004 ▶); Matsumoto *et al.* (2002 ▶); Nockemann *et al.* (2005 ▶, 2006 ▶, 2008 ▶); Reichert *et al.* (2006 ▶); Taubert (2004 ▶); Tsuda *et al.* (2001 ▶); Zhao *et al.* (2004 ▶).
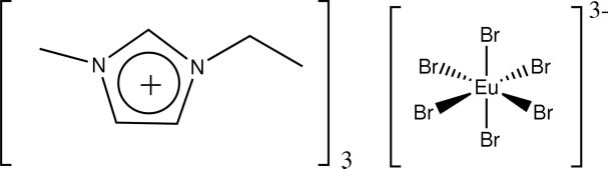


## Experimental

### 

#### Crystal data

(C_6_H_11_N_2_)_3_[EuBr_6_]
*M* *_r_* = 964.87
Monoclinic, 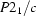

*a* = 15.765 (1) Å
*b* = 12.729 (1) Å
*c* = 14.920 (1) Å
β = 90.36 (1)°
*V* = 2994.0 (4) Å^3^
*Z* = 4
Mo *K*α radiation
μ = 10.12 mm^−1^
*T* = 100 (2) K
0.18 × 0.17 × 0.16 mm

#### Data collection

Oxford Diffraction Gemini A Ultra diffractometer
Absorption correction: multi-scan (*CrysAlis RED*; Oxford Diffraction, 2008 ▶) *T*_min_ = 0.148, *T*_max_ = 0.200
17678 measured reflections
7019 independent reflections
5043 reflections with *I* > 2σ(*I*)
*R*_int_ = 0.029

#### Refinement

*R*[*F* ^2^ > 2σ(*F* ^2^)] = 0.029
*wR*(*F* ^2^) = 0.099
*S* = 1.07
7019 reflections
290 parameters
H-atom parameters constrained
Δρ_max_ = 1.75 e Å^−3^
Δρ_min_ = −1.44 e Å^−3^

### 

Data collection: *CrysAlis CCD* (Oxford Diffraction, 2008 ▶); cell refinement: *CrysAlis RED* (Oxford Diffraction, 2008 ▶); data reduction: *CrysAlis RED*; program(s) used to solve structure: *SHELXS97* (Sheldrick, 2008 ▶); program(s) used to refine structure: *SHELXL97* (Sheldrick, 2008 ▶); molecular graphics: *DIAMOND* (Brandenburg, 2007 ▶); software used to prepare material for publication: *PLATON* (Spek, 2003 ▶).

## Supplementary Material

Crystal structure: contains datablocks I, global. DOI: 10.1107/S1600536808018382/hg2399sup1.cif

Structure factors: contains datablocks I. DOI: 10.1107/S1600536808018382/hg2399Isup2.hkl

Additional supplementary materials:  crystallographic information; 3D view; checkCIF report

## Figures and Tables

**Fig. 1.**
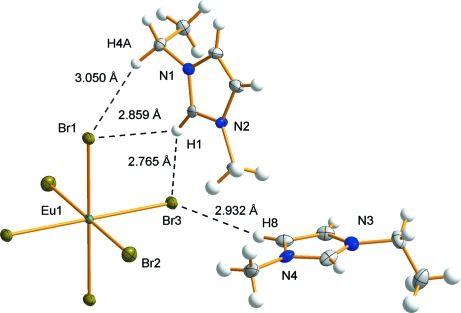
Structure of the [EuBr6]3- anion and interactions to two exemplary [EMIM]+ cations around Eu1 in the crystal structure of [EMIM]3[EuBr6]. The dashed lines indicate the hydrogen bonding interactions. Displacement ellipsoids are shown at the 50% probability level and H-atoms are drawn as small circles of arbitrary radii.

**Fig. 2.**
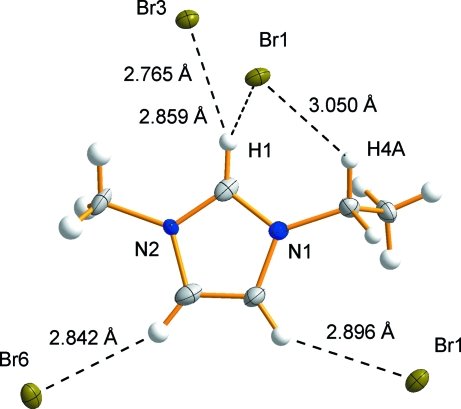
Surrounding of an [EMIM]+ cation in the crystal structure of [EMIM]3[EuBr6]. The dashed lines indicate the hydrogen bonding interactions. Displacement ellipsoids are shown at the 50% probability level and H-atoms are drawn as small circles of arbitrary radii.

**Fig. 3.**
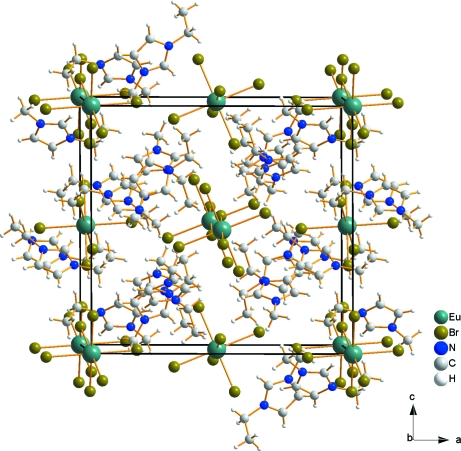
Packing of the structure of [EMIM]3[EuBr6] viewed along the b axis. The [EuBr6]3- anions are located on the corners and face-centers of the monoclinic unit cell.

## supplementary crystallographic information

### Comment

Ionic liquids are increasingly attracting the attention of inorganic and materials chemists (Taubert, 2004; Reichert *et al.*, 2006; Nockemann *et al.*, 2008). Lanthanide compounds dissolved in ionic liquids have been of interest especially due to their photoluminescence behavior (Driesen *et al.*, 2004; Binnemans, 2007; Nockemann *et al.*, 2005). Experimental and theoretical studies on lanthanide ions in halide containing imidazolium ionic liquids have been investigated regarding electrochemical and spectroscopic properties (Arenz *et al.*, 2005; Chaumont & Wipff, 2003; Tsuda *et al.*, 2001). Imidazolium cations have been reported to yield low-melting lanthanide-containing ionic liquids like [BMIM]_5_[Eu(SCN)_8_] (Nockemann *et al.*, 2006). An analogue structure to the title compound, [EMIM]_3_[LaCl_6_], has been reported previously (Matsumoto *et al.*, 2002). The title compound crystallized unexpectedly after dissolving europium bis(trifluoromethylsulfonyl)imide hexahydrate, Eu(Tf_2_N)_3_.6H_2_O in a mixture of [EMIM]Br and a nitrile functionalized imidazolium ionic liquid, 1-butyronitrile-3-methylimidazolium bis(trifluoromethylsulfonyl)imide, [C_3_CNMIM][Tf_2_N]. The crystal structure of [EMIM]_3_[EuBr_6_] (Fig. 1) consists of 1-ethyl-3-methylimidazolium cations and octahedral [EuBr_6_]^3-^ anions. The Eu—Br distances are in the range of 2.7793 (6) Å to 2.8187 (6) Å. The octahedral geometry of the two crystallographically independent [EuBr_6_]^3-^ anions is slightly distorted with the surrounding of Eu1 more distorted than Eu2 with Br—Eu—Br angles ranging from 86.90 (2)° to 93.10 (2)° for Eu1, compared to angles ranging from 89.11 (2)° to 90.89 (2)° for Eu2. All bromine anions exhibit short contacts to neighboring H-atoms of imidazolium rings ranging from 2.76 Å to 2.90 Å. All three H-atoms of each of the three crystallographically independent imidazolium cations form hydrogen bonds with bromide atoms, which is exemplarily shown in Fig. 2 for one cation. In the packing of [EMIM]_3_[EuBr_6_], the [EuBr_6_]^3-^ anions are located on the corners and face-centers of the monoclinic unit cell (Fig. 3).

### Experimental

[EMIM]_3_[EuBr_6_] crystallized unintentionally after dissolving europium(III) bis(trifluoromethylsulfonyl)imide hexahydrate, Eu(Tf_2_N)_3_.6H_2_O (0.5 g, 0.454 mmol) in a mixture of 5 ml of [EMIM]Br and 5 ml of a nitrile functionalized imidazolium ionic liquid, 1-butyronitrile-3-methylimidazolium bis(trifluoromethylsulfonyl)imide, [C_3_CNMIM][Tf_2_N]. [EMIM]Br was purchased from IoLiTec. The nitrile functionalized imidazolium ionic liquid has been synthesized following a procedure that has been reported in the literature (Zhao *et al.* 2004). The title compound crystallized as small slightly yellow blocks.

### Refinement

Hydrogen atoms were refined in the riding mode with isotropic temperature factors fixed at 1.2 times *U*_eq_ of the parent atoms (1.5 times for methyl groups).

### Crystal data

**Table:**

(C_6_H_11_N_2_)_3_[Eu_1_Br_6_]	*F*_000_ = 1824
*M**_r_* = 964.87	*D*_x_ = 2.141 Mg m^−^^3^
Monoclinic, *P*2_1_/*c*	Mo *K*α radiation λ = 0.71073 Å
Hall symbol: -P 2ybc	Cell parameters from 9203 reflections
*a* = 15.765 (1) Å	θ = 3.0–29.1º
*b* = 12.729 (1) Å	µ = 10.12 mm^−^^1^
*c* = 14.920 (1) Å	*T* = 100 (2) K
β = 90.36 (1)º	Block, yellow
*V* = 2994.0 (4) Å^3^	0.18 × 0.17 × 0.16 mm
*Z* = 4	

### Data collection

**Table:**

Oxford Diffraction Gemini A Ultra diffractometer	7019 independent reflections
Radiation source: Enhance (Mo) X-ray Source	5043 reflections with *I* > 2σ(*I*)
Monochromator: graphite	*R*_int_ = 0.029
Detector resolution: 10.3310 pixels mm^-1^	θ_max_ = 29.1º
*T* = 100(2) K	θ_min_ = 3.0º
ω and φ scans	*h* = −21→13
Absorption correction: multi-scan(CrysAlis RED; Oxford Diffraction, 2008)	*k* = −14→17
*T*_min_ = 0.148, *T*_max_ = 0.200	*l* = −19→19
17678 measured reflections	

### Refinement

**Table:**

Refinement on *F*^2^	Secondary atom site location: difference Fourier map
Least-squares matrix: full	Hydrogen site location: inferred from neighbouring sites
*R*[*F*^2^ > 2σ(*F*^2^)] = 0.029	H-atom parameters constrained
*wR*(*F*^2^) = 0.099	*w* = 1/[σ^2^(*F*_o_^2^) + (0.0541*P*)^2^] where *P* = (*F*_o_^2^ + 2*F*_c_^2^)/3
*S* = 1.07	(Δ/σ)_max_ < 0.001
7019 reflections	Δρ_max_ = 1.75 e Å^−^^3^
290 parameters	Δρ_min_ = −1.44 e Å^−^^3^
Primary atom site location: structure-invariant direct methods	Extinction correction: none

### Special details

**Table:**

Experimental. CrysAlis RED (CrysAlis RED, 2008). Empirical absorption correction using spherical harmonics, implemented in SCALE3 ABSPACK scaling algorithm.
Geometry. All e.s.d.'s (except the e.s.d. in the dihedral angle between two l.s. planes) are estimated using the full covariance matrix. The cell e.s.d.'s are taken into account individually in the estimation of e.s.d.'s in distances, angles and torsion angles; correlations between e.s.d.'s in cell parameters are only used when they are defined by crystal symmetry. An approximate (isotropic) treatment of cell e.s.d.'s is used for estimating e.s.d.'s involving l.s. planes.
Refinement. Refinement of *F*^2^ against ALL reflections. The weighted *R*-factor *wR* and goodness of fit *S* are based on *F*^2^, conventional *R*-factors *R* are based on *F*, with *F* set to zero for negative *F*^2^. The threshold expression of *F*^2^ > σ(*F*^2^) is used only for calculating *R*-factors(gt) *etc*. and is not relevant to the choice of reflections for refinement. *R*-factors based on *F*^2^ are statistically about twice as large as those based on *F*, and *R*- factors based on ALL data will be even larger.

### Fractional atomic coordinates and isotropic or equivalent isotropic displacement parameters (Å^2^)

**Table:**

	*x*	*y*	*z*	*U*_iso_*/*U*_eq_	
C1	0.9054 (4)	0.1566 (5)	0.4127 (4)	0.0200 (14)	
H1	0.9361	0.2068	0.4471	0.024*	
C2	0.8011 (5)	0.0618 (5)	0.3547 (4)	0.0257 (15)	
H2	0.7459	0.0348	0.3428	0.031*	
C3	0.8726 (4)	0.0310 (5)	0.3170 (4)	0.0228 (14)	
H3	0.8781	−0.0218	0.2725	0.027*	
C4	1.0300 (4)	0.0820 (5)	0.3349 (4)	0.0171 (13)	
H4A	1.0576	0.1500	0.3491	0.021*	
H4B	1.0379	0.0680	0.2702	0.021*	
C5	1.0725 (4)	−0.0044 (5)	0.3889 (4)	0.0212 (14)	
H5A	1.0614	0.0064	0.4528	0.032*	
H5B	1.1338	−0.0029	0.3785	0.032*	
H5C	1.0497	−0.0727	0.3703	0.032*	
C6	0.7598 (4)	0.2016 (5)	0.4680 (4)	0.0270 (16)	
H6A	0.7428	0.1604	0.5204	0.041*	
H6B	0.7098	0.2169	0.4309	0.041*	
H6C	0.7858	0.2676	0.4880	0.041*	
C7	0.6503 (4)	0.3160 (6)	0.2301 (4)	0.0235 (15)	
H7	0.5922	0.3054	0.2159	0.028*	
C8	0.7896 (4)	0.2961 (5)	0.2383 (4)	0.0243 (15)	
H8	0.8454	0.2696	0.2309	0.029*	
C9	0.7664 (4)	0.3830 (5)	0.2844 (4)	0.0217 (14)	
H9	0.8039	0.4287	0.3156	0.026*	
C10	0.6311 (5)	0.4800 (6)	0.3179 (4)	0.0306 (17)	
H10A	0.5807	0.4923	0.2794	0.037*	
H10B	0.6650	0.5455	0.3189	0.037*	
C11	0.6025 (5)	0.4553 (6)	0.4115 (5)	0.0358 (18)	
H11A	0.5715	0.3886	0.4114	0.054*	
H11B	0.5653	0.5115	0.4329	0.054*	
H11C	0.6521	0.4497	0.4512	0.054*	
C12	0.7057 (5)	0.1584 (5)	0.1507 (4)	0.0291 (16)	
H12A	0.6658	0.1111	0.1805	0.044*	
H12B	0.7607	0.1233	0.1444	0.044*	
H12C	0.6836	0.1769	0.0912	0.044*	
C13	0.7070 (4)	0.8020 (5)	0.3480 (4)	0.0272 (15)	
H13	0.6671	0.8381	0.3841	0.033*	
C14	0.8247 (5)	0.7279 (7)	0.3036 (5)	0.042 (2)	
H14	0.8813	0.7024	0.3025	0.051*	
C15	0.7644 (4)	0.7205 (7)	0.2371 (5)	0.037 (2)	
H15	0.7720	0.6888	0.1800	0.045*	
C16	0.6146 (5)	0.7797 (6)	0.2138 (5)	0.0376 (18)	
H16A	0.5698	0.8121	0.2508	0.045*	
H16B	0.5942	0.7100	0.1937	0.045*	
C17	0.6318 (5)	0.8486 (6)	0.1327 (5)	0.045 (2)	
H17A	0.6572	0.9150	0.1524	0.068*	
H17B	0.5783	0.8629	0.1011	0.068*	
H17C	0.6709	0.8123	0.0923	0.068*	
C18	0.8275 (5)	0.8070 (6)	0.4594 (4)	0.0316 (17)	
H18A	0.7897	0.8504	0.4959	0.047*	
H18B	0.8800	0.8458	0.4476	0.047*	
H18C	0.8410	0.7421	0.4917	0.047*	
N1	0.9384 (3)	0.0896 (4)	0.3541 (3)	0.0166 (11)	
N2	0.8220 (3)	0.1409 (4)	0.4148 (3)	0.0141 (10)	
N3	0.6826 (4)	0.3942 (4)	0.2789 (3)	0.0281 (13)	
N4	0.7163 (3)	0.2543 (4)	0.2045 (3)	0.0194 (11)	
N5	0.6912 (4)	0.7670 (4)	0.2673 (4)	0.0294 (13)	
N6	0.7846 (4)	0.7809 (4)	0.3727 (4)	0.0273 (13)	
Br1	0.99929 (4)	0.37839 (5)	0.34343 (4)	0.01889 (14)	
Br2	0.82537 (4)	0.52642 (5)	0.48677 (4)	0.02342 (15)	
Br3	1.03396 (4)	0.68086 (5)	0.39729 (4)	0.01711 (14)	
Br4	0.33611 (4)	0.02950 (5)	0.43034 (4)	0.01855 (14)	
Br5	0.50481 (4)	0.21190 (5)	0.54969 (4)	0.02105 (14)	
Br6	0.56634 (4)	0.05083 (5)	0.33129 (4)	0.02125 (15)	
Eu1	1.0000	0.5000	0.5000	0.00923 (9)	
Eu2	0.5000	0.0000	0.5000	0.01018 (10)	

### Atomic displacement parameters (Å^2^)

**Table:**

	*U*^11^	*U*^22^	*U*^33^	*U*^12^	*U*^13^	*U*^23^
C1	0.031 (4)	0.013 (3)	0.015 (3)	−0.001 (3)	0.008 (3)	0.003 (2)
C2	0.036 (4)	0.017 (3)	0.024 (3)	−0.003 (3)	0.003 (3)	−0.004 (3)
C3	0.025 (3)	0.018 (3)	0.026 (3)	0.000 (3)	0.004 (3)	−0.007 (3)
C4	0.017 (3)	0.021 (3)	0.014 (3)	−0.001 (3)	0.000 (2)	0.000 (3)
C5	0.014 (3)	0.021 (3)	0.029 (3)	0.001 (3)	−0.003 (3)	0.002 (3)
C6	0.036 (4)	0.018 (3)	0.028 (3)	0.005 (3)	0.015 (3)	−0.005 (3)
C7	0.016 (3)	0.039 (4)	0.015 (3)	0.000 (3)	−0.006 (2)	0.004 (3)
C8	0.028 (4)	0.026 (4)	0.019 (3)	−0.004 (3)	−0.001 (3)	0.005 (3)
C9	0.023 (3)	0.026 (4)	0.017 (3)	−0.005 (3)	−0.008 (3)	0.002 (3)
C10	0.036 (4)	0.028 (4)	0.028 (4)	0.013 (3)	−0.003 (3)	0.002 (3)
C11	0.026 (4)	0.047 (5)	0.035 (4)	−0.003 (3)	0.006 (3)	−0.009 (4)
C12	0.043 (4)	0.023 (4)	0.021 (3)	−0.005 (3)	0.009 (3)	−0.004 (3)
C13	0.029 (4)	0.027 (4)	0.026 (3)	−0.001 (3)	0.000 (3)	−0.007 (3)
C14	0.050 (5)	0.054 (6)	0.023 (4)	0.023 (4)	0.000 (3)	−0.014 (4)
C15	0.014 (3)	0.063 (6)	0.035 (4)	0.015 (3)	−0.005 (3)	−0.014 (4)
C16	0.029 (4)	0.037 (5)	0.047 (5)	0.004 (3)	−0.009 (3)	−0.017 (4)
C17	0.054 (5)	0.044 (5)	0.037 (4)	0.000 (4)	−0.026 (4)	−0.005 (4)
C18	0.036 (4)	0.034 (4)	0.025 (3)	0.012 (3)	−0.007 (3)	−0.008 (3)
N1	0.017 (3)	0.016 (3)	0.017 (2)	−0.002 (2)	0.001 (2)	−0.002 (2)
N2	0.009 (2)	0.017 (3)	0.016 (2)	−0.001 (2)	0.0001 (19)	−0.004 (2)
N3	0.044 (4)	0.021 (3)	0.019 (3)	0.003 (3)	0.006 (3)	0.000 (2)
N4	0.019 (3)	0.026 (3)	0.014 (2)	−0.004 (2)	0.002 (2)	0.001 (2)
N5	0.030 (3)	0.023 (3)	0.036 (3)	−0.004 (3)	0.005 (3)	−0.007 (3)
N6	0.034 (3)	0.021 (3)	0.027 (3)	0.010 (3)	0.008 (3)	0.000 (2)
Br1	0.0281 (3)	0.0149 (3)	0.0137 (3)	−0.0037 (3)	0.0016 (2)	−0.0014 (2)
Br2	0.0153 (3)	0.0261 (4)	0.0288 (3)	0.0010 (3)	−0.0015 (3)	−0.0004 (3)
Br3	0.0229 (3)	0.0138 (3)	0.0146 (3)	−0.0018 (2)	−0.0001 (2)	0.0010 (2)
Br4	0.0137 (3)	0.0225 (3)	0.0195 (3)	0.0012 (2)	−0.0022 (2)	0.0004 (3)
Br5	0.0235 (3)	0.0168 (3)	0.0228 (3)	−0.0011 (3)	−0.0039 (2)	0.0003 (3)
Br6	0.0185 (3)	0.0301 (4)	0.0152 (3)	−0.0005 (3)	0.0030 (2)	0.0040 (3)
Eu1	0.01068 (18)	0.00812 (19)	0.00889 (18)	−0.00080 (16)	−0.00038 (14)	0.00018 (15)
Eu2	0.00931 (18)	0.0124 (2)	0.00889 (18)	0.00017 (16)	0.00048 (14)	0.00114 (16)

### Geometric parameters (Å, °)

**Table:**

C1—N1	1.329 (7)	C12—H12A	0.9800
C1—N2	1.331 (8)	C12—H12B	0.9800
C1—H1	0.9500	C12—H12C	0.9800
C2—C3	1.322 (9)	C13—N6	1.304 (9)
C2—N2	1.388 (8)	C13—N5	1.306 (8)
C2—H2	0.9500	C13—H13	0.9500
C3—N1	1.390 (8)	C14—C15	1.373 (10)
C3—H3	0.9500	C14—N6	1.387 (8)
C4—N1	1.478 (7)	C14—H14	0.9500
C4—C5	1.517 (8)	C15—N5	1.375 (8)
C4—H4A	0.9900	C15—H15	0.9500
C4—H4B	0.9900	C16—N5	1.452 (9)
C5—H5A	0.9800	C16—C17	1.520 (11)
C5—H5B	0.9800	C16—H16A	0.9900
C5—H5C	0.9800	C16—H16B	0.9900
C6—N2	1.482 (7)	C17—H17A	0.9800
C6—H6A	0.9800	C17—H17B	0.9800
C6—H6B	0.9800	C17—H17C	0.9800
C6—H6C	0.9800	C18—N6	1.495 (9)
C7—N3	1.332 (8)	C18—H18A	0.9800
C7—N4	1.360 (8)	C18—H18B	0.9800
C7—H7	0.9500	C18—H18C	0.9800
C8—C9	1.355 (9)	Br1—Eu1	2.8024 (6)
C8—N4	1.365 (8)	Br2—Eu1	2.7793 (6)
C8—H8	0.9500	Br3—Eu1	2.8188 (6)
C9—N3	1.330 (8)	Br4—Eu2	2.8041 (6)
C9—H9	0.9500	Br5—Eu2	2.7982 (6)
C10—N3	1.483 (8)	Br6—Eu2	2.8074 (5)
C10—C11	1.503 (9)	Eu1—Br2^i^	2.7794 (6)
C10—H10A	0.9900	Eu1—Br1^i^	2.8023 (6)
C10—H10B	0.9900	Eu1—Br3^i^	2.8187 (6)
C11—H11A	0.9800	Eu2—Br5^ii^	2.7982 (6)
C11—H11B	0.9800	Eu2—Br4^ii^	2.8041 (6)
C11—H11C	0.9800	Eu2—Br6^ii^	2.8073 (5)
C12—N4	1.470 (8)		
			
N1—C1—N2	108.1 (5)	C17—C16—H16A	109.6
N1—C1—H1	125.9	N5—C16—H16B	109.6
N2—C1—H1	125.9	C17—C16—H16B	109.6
C3—C2—N2	106.8 (6)	H16A—C16—H16B	108.1
C3—C2—H2	126.6	C16—C17—H17A	109.5
N2—C2—H2	126.6	C16—C17—H17B	109.5
C2—C3—N1	108.0 (6)	H17A—C17—H17B	109.5
C2—C3—H3	126.0	C16—C17—H17C	109.5
N1—C3—H3	126.0	H17A—C17—H17C	109.5
N1—C4—C5	111.9 (5)	H17B—C17—H17C	109.5
N1—C4—H4A	109.2	N6—C18—H18A	109.5
C5—C4—H4A	109.2	N6—C18—H18B	109.5
N1—C4—H4B	109.2	H18A—C18—H18B	109.5
C5—C4—H4B	109.2	N6—C18—H18C	109.5
H4A—C4—H4B	107.9	H18A—C18—H18C	109.5
C4—C5—H5A	109.5	H18B—C18—H18C	109.5
C4—C5—H5B	109.5	C1—N1—C3	108.2 (5)
H5A—C5—H5B	109.5	C1—N1—C4	123.8 (5)
C4—C5—H5C	109.5	C3—N1—C4	128.0 (5)
H5A—C5—H5C	109.5	C1—N2—C2	108.9 (5)
H5B—C5—H5C	109.5	C1—N2—C6	126.3 (5)
N2—C6—H6A	109.5	C2—N2—C6	124.7 (5)
N2—C6—H6B	109.5	C9—N3—C7	109.3 (5)
H6A—C6—H6B	109.5	C9—N3—C10	126.9 (6)
N2—C6—H6C	109.5	C7—N3—C10	123.8 (6)
H6A—C6—H6C	109.5	C7—N4—C8	108.5 (5)
H6B—C6—H6C	109.5	C7—N4—C12	123.2 (6)
N3—C7—N4	107.1 (5)	C8—N4—C12	128.2 (6)
N3—C7—H7	126.4	C13—N5—C15	107.1 (6)
N4—C7—H7	126.4	C13—N5—C16	128.4 (6)
C9—C8—N4	106.0 (6)	C15—N5—C16	124.3 (6)
C9—C8—H8	127.0	C13—N6—C14	108.8 (6)
N4—C8—H8	127.0	C13—N6—C18	128.1 (5)
N3—C9—C8	109.1 (6)	C14—N6—C18	123.1 (6)
N3—C9—H9	125.5	Br2—Eu1—Br2^i^	180.0
C8—C9—H9	125.5	Br2—Eu1—Br1^i^	89.496 (19)
N3—C10—C11	112.2 (6)	Br2^i^—Eu1—Br1^i^	90.505 (18)
N3—C10—H10A	109.2	Br2—Eu1—Br1	90.505 (19)
C11—C10—H10A	109.2	Br2^i^—Eu1—Br1	89.494 (18)
N3—C10—H10B	109.2	Br1^i^—Eu1—Br1	180.0
C11—C10—H10B	109.2	Br2—Eu1—Br3^i^	86.905 (18)
H10A—C10—H10B	107.9	Br2^i^—Eu1—Br3^i^	93.095 (18)
C10—C11—H11A	109.5	Br1^i^—Eu1—Br3^i^	89.872 (16)
C10—C11—H11B	109.5	Br1—Eu1—Br3^i^	90.129 (16)
H11A—C11—H11B	109.5	Br2—Eu1—Br3	93.096 (18)
C10—C11—H11C	109.5	Br2^i^—Eu1—Br3	86.904 (18)
H11A—C11—H11C	109.5	Br1^i^—Eu1—Br3	90.128 (16)
H11B—C11—H11C	109.5	Br1—Eu1—Br3	89.871 (16)
N4—C12—H12A	109.5	Br3^i^—Eu1—Br3	180.0
N4—C12—H12B	109.5	Br5—Eu2—Br5^ii^	180.0
H12A—C12—H12B	109.5	Br5—Eu2—Br4^ii^	90.431 (18)
N4—C12—H12C	109.5	Br5^ii^—Eu2—Br4^ii^	89.569 (18)
H12A—C12—H12C	109.5	Br5—Eu2—Br4	89.568 (18)
H12B—C12—H12C	109.5	Br5^ii^—Eu2—Br4	90.432 (18)
N6—C13—N5	111.3 (6)	Br4^ii^—Eu2—Br4	180.0
N6—C13—H13	124.4	Br5—Eu2—Br6^ii^	89.668 (18)
N5—C13—H13	124.4	Br5^ii^—Eu2—Br6^ii^	90.332 (18)
C15—C14—N6	104.7 (7)	Br4^ii^—Eu2—Br6^ii^	89.105 (17)
C15—C14—H14	127.6	Br4—Eu2—Br6^ii^	90.895 (17)
N6—C14—H14	127.6	Br5—Eu2—Br6	90.331 (18)
C14—C15—N5	108.2 (6)	Br5^ii^—Eu2—Br6	89.668 (18)
C14—C15—H15	125.9	Br4^ii^—Eu2—Br6	90.896 (17)
N5—C15—H15	125.9	Br4—Eu2—Br6	89.104 (17)
N5—C16—C17	110.5 (6)	Br6^ii^—Eu2—Br6	180.0
N5—C16—H16A	109.6		

Symmetry codes: (i) −*x*+2, −*y*+1, −*z*+1; (ii) −*x*+1, −*y*, −*z*+1.
